# Clinically focused multi-cohort benchmarking as a tool for external validation of artificial intelligence algorithm performance in basic chest radiography analysis

**DOI:** 10.1038/s41598-022-16514-7

**Published:** 2022-07-27

**Authors:** Jan Rudolph, Balthasar Schachtner, Nicola Fink, Vanessa Koliogiannis, Vincent Schwarze, Sophia Goller, Lena Trappmann, Boj F. Hoppe, Nabeel Mansour, Maximilian Fischer, Najib Ben Khaled, Maximilian Jörgens, Julien Dinkel, Wolfgang G. Kunz, Jens Ricke, Michael Ingrisch, Bastian O. Sabel, Johannes Rueckel

**Affiliations:** 1 Department of Radiology, University Hospital, LMU Munich, Marchioninistr. 15, 81377 Munich, Germany; 2 Comprehensive Pneumology Center, German Center for Lung Research, Munich, Germany; 3 Department of Medicine I, University Hospital, LMU Munich, Munich, Germany; 4 Department of Medicine II, University Hospital, LMU Munich, Munich, Germany; 5 Department of Orthopaedics and Trauma Surgery, Musculoskeletal University Center Munich (MUM), University Hospital, LMU Munich, Munich, Germany; 6 Department of Radiology, Asklepios Fachklinik München, Gauting, Germany; 7 Institute of Neuroradiology, University Hospital, LMU Munich, Munich, Germany

**Keywords:** Computational science, Scientific data, Respiratory tract diseases, Diagnosis, Clinical trial design

## Abstract

Artificial intelligence (AI) algorithms evaluating [supine] chest radiographs ([S]CXRs) have remarkably increased in number recently. Since training and validation are often performed on subsets of the same overall dataset, external validation is mandatory to reproduce results and reveal potential training errors. We applied a multicohort benchmarking to the publicly accessible (S)CXR analyzing AI algorithm CheXNet, comprising three clinically relevant study cohorts which differ in patient positioning ([S]CXRs), the applied reference standards (CT-/[S]CXR-based) and the possibility to also compare algorithm classification with different medical experts’ reading performance. The study cohorts include [1] a cohort, characterized by 563 CXRs acquired in the emergency unit that were evaluated by 9 readers (radiologists and non-radiologists) in terms of 4 common pathologies, [2] a collection of 6,248 SCXRs annotated by radiologists in terms of pneumothorax presence, its size and presence of inserted thoracic tube material which allowed for subgroup and confounding bias analysis and [3] a cohort consisting of 166 patients with SCXRs that were evaluated by radiologists for underlying causes of basal lung opacities, all of those cases having been correlated to a timely acquired computed tomography scan (SCXR and CT within < 90 min). CheXNet non-significantly exceeded the radiology resident (RR) consensus in the detection of suspicious lung nodules (cohort [1], AUC AI/RR: 0.851/0.839, *p* = 0.793) and the radiological readers in the detection of basal pneumonia (cohort [3], AUC AI/reader consensus: 0.825/0.782, *p* = 0.390) and basal pleural effusion (cohort [3], AUC AI/reader consensus: 0.762/0.710, *p* = 0.336) in SCXR, partly with AUC values higher than originally published (“Nodule”: 0.780, “Infiltration”: 0.735, “Effusion”: 0.864). The classifier “Infiltration” turned out to be very dependent on patient positioning (best in CXR, worst in SCXR). The pneumothorax SCXR cohort [2] revealed poor algorithm performance in CXRs without inserted thoracic material and in the detection of small pneumothoraces, which can be explained by a known systematic confounding error in the algorithm training process. The benefit of clinically relevant external validation is demonstrated by the differences in algorithm performance as compared to the original publication. Our multi-cohort benchmarking finally enables the consideration of confounders, different reference standards and patient positioning as well as the AI performance comparison with differentially qualified medical readers.

## Introduction

In primary diagnostics, [supine] chest radiography ([S]CXR), performed for common indications such as suspected pneumonia, pneumothorax, effusion, verification of catheter location, and/or detection of pulmonary nodules, remains one of the most frequently requested examinations worldwide, with significant public health implications^1–5^. Image interpretation is often aggravated by projection phenomena, requires a high level of experience and remains challenging for radiologists as well as a for non-radiologists^6–9^.

During the past years, clinical applications of artificial intelligence (AI) algorithms have been increasingly brought into scientific focus since several AI systems have already successfully mimicked healthcare specialists’ diagnostic performance levels^10–18^. A considerable number of CXR interpreting algorithms is trained on the basis of publicly available data sets with labels extracted from radiology reports using natural language processing (NLP)^19,20^, with algorithms are commonly validated on subgroups of these data sets. To identify potential confounders, external benchmarking is of exceptional importance to the algorithm training development process.

In the current paper, we present an external benchmarking pipeline that comprises three different (S)CXR cohorts. The cohorts differ in patient positioning during image acquisition (supine vs. upright), the underlying reference standards (radiologists’ [S]CXR vs. CT labelling) and the possibility to compare algorithm classification with different medical experts’ performances. By combining the cohorts, we try to cover a variety of different scenarios of daily clinical practice. Based on the cohorts, we characterize the performance of a well-established and publicly available implementation of the AI algorithm CheXNet^21–23^. By comparing the performance results with the results of the original publication, we demonstrate the necessity of extensive external algorithm validation including the analysis based on different cohort subgroups.

## Materials and methods

Approval of the institutional ethics commission (Ethics Committee of the Medical Faculty of Ludwig-Maximilians-University Munich) was obtained for this study (approval numbers 418-16, 18-399 and 19-541). Informed consent was waived due to the retrospective character of the study by the institutional ethics commission (Ethics Committee of the Medical Faculty of Ludwig-Maximilians-University Munich). All methods were performed in accordance with the relevant guidelines and regulations of Nature Research journals.

### Patient cohorts (image selection and reading)

In the following paragraphs we display the three different (S)CXR cohorts.

#### Emergency unit chest radiograph cohort (CXR EU)

**Figure 1:**
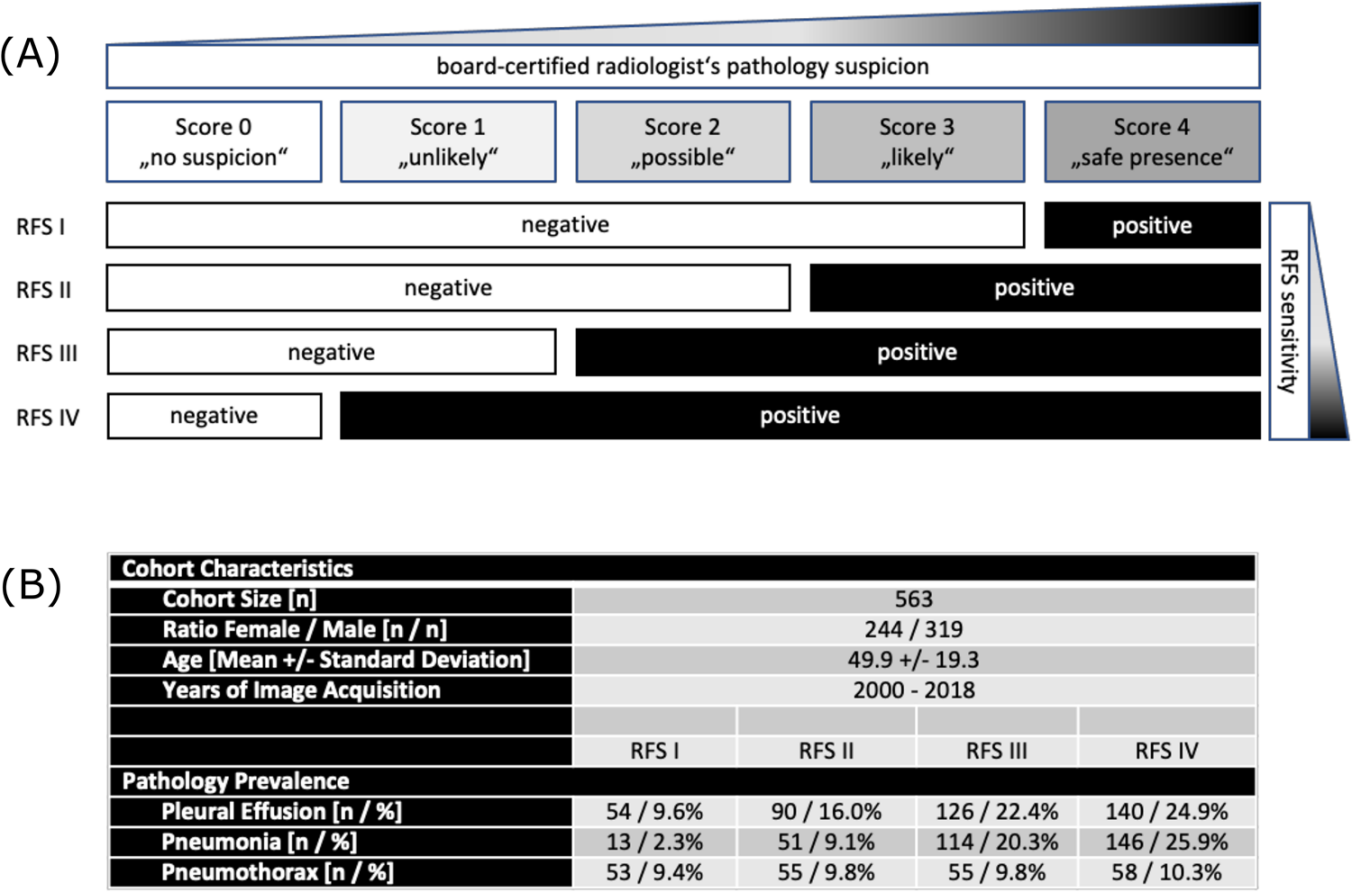
Reference standard and characteristics in the CXR EU cohort; (**A**) illustrates the definition of reference standards based on suspicion scores of the board-certified radiologists. According to the principle of a majority voting 4 reference standards (RFS) were built—RFS I being the most specific and RFS IV being the most sensitive one; (**B**) illustrates cohorts characteristics and pathology prevalence according to RFS I–IV.

Cohort containing a total of 563 CXRs in upright position and posterior-anterior (PA) projection that were exclusively acquired in the emergency unit (EU) and were independently evaluated by 9 medical readers of different diagnostic expertise including radiologists (board-certified radiologists [BCRs], radiology residents [RRs]) and non-radiologists (non-radiology residents [NRRs]) (Fig. 1). CXRs contain a representative composition of common findings in the EU: Images without suspected pathologies, pleural effusions, pneumothoraces, consolidations suspicious for pneumonia and lung lesions. We defined the four target diseases as common, clinically important thoracic diseases of emergency radiology for which the primary diagnosis is usually made by chest radiography and for which rapid further therapy/diagnosis is required. Together, they cover a majority of the non-cardiac, non-traumatic causes of acute chest pain visible in the CXR^24^. With an estimated and/or approximated incidence of 1.5–14.0 (pneumonia)^25^, up to 322.7 (pleural effusion)^26^, 22.7 (pneumothorax)^27^ and 6.6–12.6 per 100,000 patients per year (pulmonary nodules)^28^, the four pathologies occur very frequently. In fact, pulmonary malignant neoplasms and pneumonia are among the top five respiratory diseases in terms of global burden^29^. A detailed cohort description is provided by Rudolph et al.^30^.

Readers had to evaluate the images in terms of the mentioned findings on a five-point Likert scale: 0—no suspicion/1—unlikely/2—possible/3—likely/4—safe presence. BCR’s reading served as reference standard (RFS) exclusively. The consensus of all three BCR readers was converted into yes-or-no-call RFSs of different sensitivity/specificity as follows: Likert choices 0–3 have been pooled and considered as negative to build the most specific RFS I, choices 1–4 have been pooled and considered as positive representing the most sensitive RFS IV. The other RFSs (II/III) were built accordingly. The scheme is illustrated in Fig. 1A. The final RFSs were built by consensus (majority voting) based on the individual BCR’s yes-or-no-calls. The resulting pathology prevalences depending on the final RFSs are illustrated in Fig. 1B. Based on the above mentioned RFS, the performances of other readers (RRs and NNRs) have been compared with algorithm performance.

#### Supine chest radiograph unilateral pneumothorax cohort (SCXR PTX)

Cohort containing a total of 6,258 supine CXR (SCXR) cases in anterior–posterior view (AP) that were annotated in terms of a present unilateral pneumothorax (PTX) including the measure of dehiscence and the presence of a thoracic tube (Table 1). Detailed cohort information is described by Rueckel et al.^31^.

**Table 1:** Characteristics of the SCXR PTX cohort. The table shows the absolute and relative quantities of the subgroups that are covered in the cohort. The pneumothorax group is furthermore subdivided in terms of the measured maximal pleural dehiscence.

	Thoracic tube inserted		Sum
	Yes	No	
**Unilateral PTX (n = 1476)**			
(A) Dehiscence < 1 cm	446 (72.4%)	170 (27.6%)	616 (41.7%)
(B) Dehiscence 1–2 cm	341 (76.1%)	107 (23.9%)	448 (30.5%)
(C) Dehiscence > 2 cm	285 (71.6%)	117 (28.4%)	412 (27.8%)
Sum	1082 (73.3%)	394 (26.7%)	1476 (100.0%)
**Healthy controls (n = 4782)**			
PTX-negative	627 (13.1%)	4155 (86.9%)	4782 (100.0%)

The identified images were annotated by two well-trained fourth-year medical students (the first approximately 50 cases were directly supervised by a radiology resident [RR]). Questionable cases (approximately 10–20%) were marked for review by a RR with 3 years of experience in thoracic imaging. Images have been annotated for PTX presence, PTX size (maximal dehiscence of visceral pleura from the thoracic wall, subgroups according to < 1 cm, 1–2 cm, > 2 cm) and inserted thoracic tubes. A total of 1476 cases with unilateral PTX and 4782 PTX negative control cases were identified, see Table 1.

#### Supine chest radiograph basal lung opacities in critically ill patients (SCXR BLO)

Cohort with a total of 166 patients who received both, an SCXR image in AP view and a CT scan (at least including the basal lung zones) within 90 min without any intervention in between. The cohort is used to differentiate basal lung opacities on SCXR, which are usually difficult to interpret for human readers. Due to the short time interval between SCXR and CT imaging with appropriate clinical indications for reapplication of radiation, there is a shift in this data set to critically ill patients, which predicts pathologies considered in the context of common causes of critical airway disease that are difficult to detect with SCXR alone. Detailed cohort characteristics are described in Kunz et al.^32^ and Rueckel et al.^14^.

SCXR images were evaluated by two radiological readers (1 BCR and 1 RR with 6 months of experience in thoracic imaging interpretation) regarding suspected pneumonia. Suspicion was side-separately quantified based on a three-point Likert scale: 0—no pneumonia, 1—possible pneumonia and 2—highly suspected pneumonia. The readers were blinded to the CT data. A consensus of both reading results was formed (in case no consensus could be reached BCR’s decision was considered). In a second reading process, another BCR (also blinded to CT data) evaluated the SCXRs images side-separately for the presence of 0—no pleural effusion, 1—possible pleural effusion and 2—highly suspected pleural effusion. To get a binary decision output for further statistical analysis, suspicion scores were pooled as follows: 1 and 2 were pooled as positive for pneumonia/pleural effusion, representing a sensitive reading; 0 and 1 were pooled and considered to be negative, representing a specific reading. CT scans served as RFS to distinguish consolidations suspicious for pneumonia and pleural effusions from other reasons for basal lung opacities (CT readers were blinded to all clinical information and SCXR results). Quantities of positive cases for pneumonia and/or pleural effusion are shown in Table 2.

**Table 2:** Characteristics of the SCXR BLO cohort. The table shows the absolute and relative quantities of the subgroups that are covered in the cohort. The pneumothorax group is furthermore subdivided in terms of the measured maximal pleural dehiscence.

	Unilateral	Bilateral	Sum
Pneumonia	17 (32.7%)	35 (67.3%)	52 (31.3%)
No pneumonia	–	–	114 (68.7%)
Pleural effusion	28 (27.2%)	75 (72.8%)	103 (62.0%)
No pleural effusion	–	–	63 (38.0%)

### Artificial intelligence algorithm

Benchmarking was performed on the convolutional neural network CheXNet (“AI_CheXNet”) that aims to mimic or outperform radiologist’s performance levels, was trained and validated on ChestX-ray14 dataset ^20^ and originally introduced by Rajpurkar et al.^21,22^. We used the open-to-public Python implementation by arnoweng from GitHub.com^23^. As required by the algorithm, DICOM files were converted into PNG format using the Python Library “cv2” (version 4.5.1). All DICOMs were controlled to be saved in negative mode (“bones white”) before conversion to PNG format using “skimage” (version 0.18.1). For those images with DICOM tags “WindowWidth” and “WindowCenter” available, intensities in the range of WindowCenter ± WindowsCenter/2 were compressed to 8-bit and scaled to the range 0 to 255 using “rescale_intensity” from skimage. For all other images, intensities were rescaled from the range of maximum/minimum intensity to 8-bit.

### Results quantification and statistical analysis

AI algorithm and reading performance was quantified using receiver operator characteristics (ROC) analysis and calculation of the area under the ROC curve (AUC). Optimized ROC operating points were approximated to the maximum sum of sensitivity and specificity (Youden’s J Statistics) and marked with dots in the corresponding ROC-curves. Diagnostic metrics for the optimized operating points (accuracy [acc], sensitivity [sens], specificity [spec], positive [ppv] and negative predictive values [npv], false positive rate [fpr], false negative rate [fnr]) were calculated and tabularly illustrated. All statistics and graphic illustrations have been performed using open-source programming language R^33^.

## Results

### Pneumothorax (PTX)

**Figure 2:**
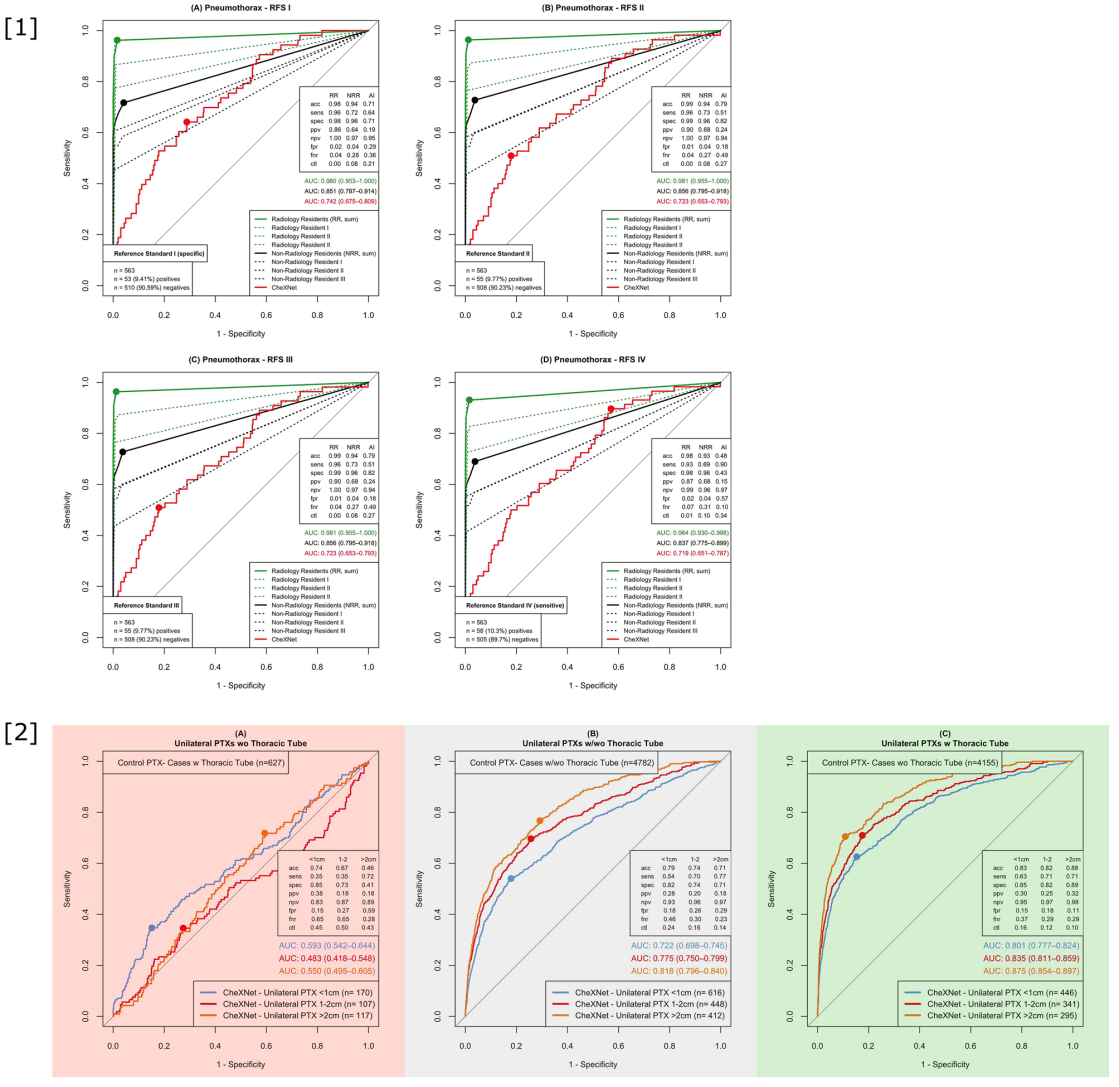
Benchmarking of pneumothorax detection; [**1**] shows the results of the CXR EU cohort for all reference standards (RFS I–IV, (**A**)–(**D**)) pooled in subgroups of Radiology Residents (RR), Non-Radiology Residents (NRR) and CheXNet classifier “Pneumothorax” – CheXNet classifier “Pneumothorax” was statistically significant outperformed by RR consensus (green curve) and performed worse than NRR consensus (black curve) in all four RFS. Dotted green and black lines show individual reader’s performance levels. Accuracy (acc), sensitivity (sens), specificity (spec), positive predictive value (ppv), negative predictive value (npv), false positive rate (fpr), false negative rate (fnr), close top left (ctl) and area under the curve (AUC; with 95%-confidence intervals) are given for all consensus and CheXNet AI ROCs; [**2**] shows benchmarking results of the SCXR PTX cohort pooled by unilateral PTX size. (**A**) Considering PTX positive cases without inserted thoracic tubes CheXNet performed poorly in all subgroups. (**B**/**C**) Performance increases notably if the proportion of cases with inserted thoracic tube increases (**B**) or if PTX positive cases with inserted thoracic tubes are considered exclusively (**C**).

Our benchmark pipeline can test the AI pneumothorax detection rate in the CXR EU cohort (Fig. 2—[1]) and the SCXR PTX cohort (Fig. 2—[2]).

The CXR EU cohort allows for a direct performance comparison to radiology residents and non-radiology residents on PA CXR that are free of any foreign material (e. g. thoracic tubes). ROC curves are based on the four different RFSs (I–IV, see “Materials and methods” section). In Fig. 2—[1] the red line represents the AI algorithm, the green line the RR consensus (sum of the three individual RR readers) and the black line the NRR consensus (sum of the three individual NRR readers)—individual reader performance is illustrated by the dotted lines. Since pneumothorax detection was basically a yes-or-no-call for the readers, intermediate reading scores were disproportionally underrepresented (reading scores 1, 2 and 3 made up only 0.71% of all answers). The four different ROC curves (Fig. 2—[1], (A)–(D)) therefore show no major differences. For the clinically most relevant RFS IV (most sensitive) CheXNet AI AUC was 0.719, compared to RR consensus AUC 0.964 and NRR consensus AUC 0.837, which means that the AI algorithm performance was exceeded by NRRs (*p* < 0.05) and outperformed by RRs reader consensus for pneumothorax detection (*p* < 0.001).

Our SCXR PTX cohort allows for a further evaluation of PTXs depending on size and the presence of thoracic tubes and can additionally give a hint towards performance in SCXR which are more difficult to analyze. Note that corresponding CheXNet performances have already been published ^31,^ but the key results are now uniformly illustrated according to our multi-cohort benchmarking: CheXNet showed major loss of performance in distinguishing PTX positive cases without thoracic tubes (TT) from PTX negative cases with inserted TT (Fig. 2—[2] (A); maximum AUC: 0.593), but had a better performance in detecting PTX positive cases with inserted thoracic tubes among PTX negative cases without thoracic tubes inserted (Fig. 2—[2] (C); maximum AUC: 0.875). This demonstrates the strong effect of confounding thoracic tubes on algorithm performance. Considering all cases, performance was best for bigger PTXs (Fig. 2—[2] (B); PTX > 2 cm AUC: 0.818), but the detection accuracy even for the largest PTX sizes was completely eliminated by the TT-related confounding bias represented by AUCs not significantly exceeding 0.5 (Fig. 2—[2] (A); PTX < 1 cm AUC: 0.593 (95% confidence interval [CI]: 0.542 – 0.644), PTX 1 – 2 cm AUC: 0.483 (CI 0.418–0.548), PTX > 2 cm AUC: 0.550 (CI 0.495–0.605)). For comparison with the performance results of CheXNet as mentioned in the original publication (AUCs) see Table 3.

**Table 3:** Benchmarking performance compared to originally published performance results. The table shows a comparison of the benchmarking cohort performance results (AUCs) with 95% confidence intervals (CI) compared to the AUCs of the original publication; PTX pos (neg) w (w/o) TT: pneumothorax positive (negative) with (without) inserted thoracic tube; Pot. malignant: potentially malignant

CheXNet classifier	Benchmarking cohorts (AUCs)	Original Publication ^21^ (AUCs)
Pneumothorax	CXR EU cohort: 0.719 (CI: 0.651–0.787) [Fig. 2, 1D] SCXR PTX cohort PTX pos w/o TT vs PTX neg w TT : 0.483–0.593 [Fig. 2, 2A] PTX pos w TT vs PTX neg w/o TT: 0.801–0.875 [Fig. 2, 2B] All cases: 0.722–0.818 [Fig. 2, 2C]	0.889
Effusion	CXR EU cohort: 0.897 (CI: 0.866–0.928) [Fig. 3, 1D] SCXR BLO cohort: 0.762 (CI: 0.687–0.837) [Fig. 3, 3B]	0.864
Consolidation	CXR EU cohort: 0.873 (CI: 0.841–0.905) [Fig. 3, 2D] SCXR BLO cohort: 0.673 (CI: 0.592–0.754) [Fig. 3, 3A]	0.790
Infiltration	CXR EU cohort: 0.737 (CI: 0.684–0.790) [Fig. 3, 2D] SCXR BLO cohort: 0.825 (CI: 0.761–0.889) [Fig. 3, 3A]	0.735
Pneumonia	CXR EU cohort: 0.876 (CI: 0.844–0.908) [Fig. 3, 2D] SCXR BLO cohort: 0.801 (CI: 0.734–0.869) [Fig. 3, 3A]	0.768
Nodule	CXR EU cohort: All nodules: 0.785 (0.728–0.842) [Fig. 4, A4] Pot. malignant 0.851 (0.790–0.912) [Fig. 4, B4]	0.780
Mass	CXR EU cohort: All nodules: 0.750 (0.703–0.798) [Fig. 4, A4] Pot. malignant: 0.800 (0.738–0.862) [Fig. 4, B4]	0.868

### Pleural effusion and consolidations suspicious of pneumonia

**Figure 3:**
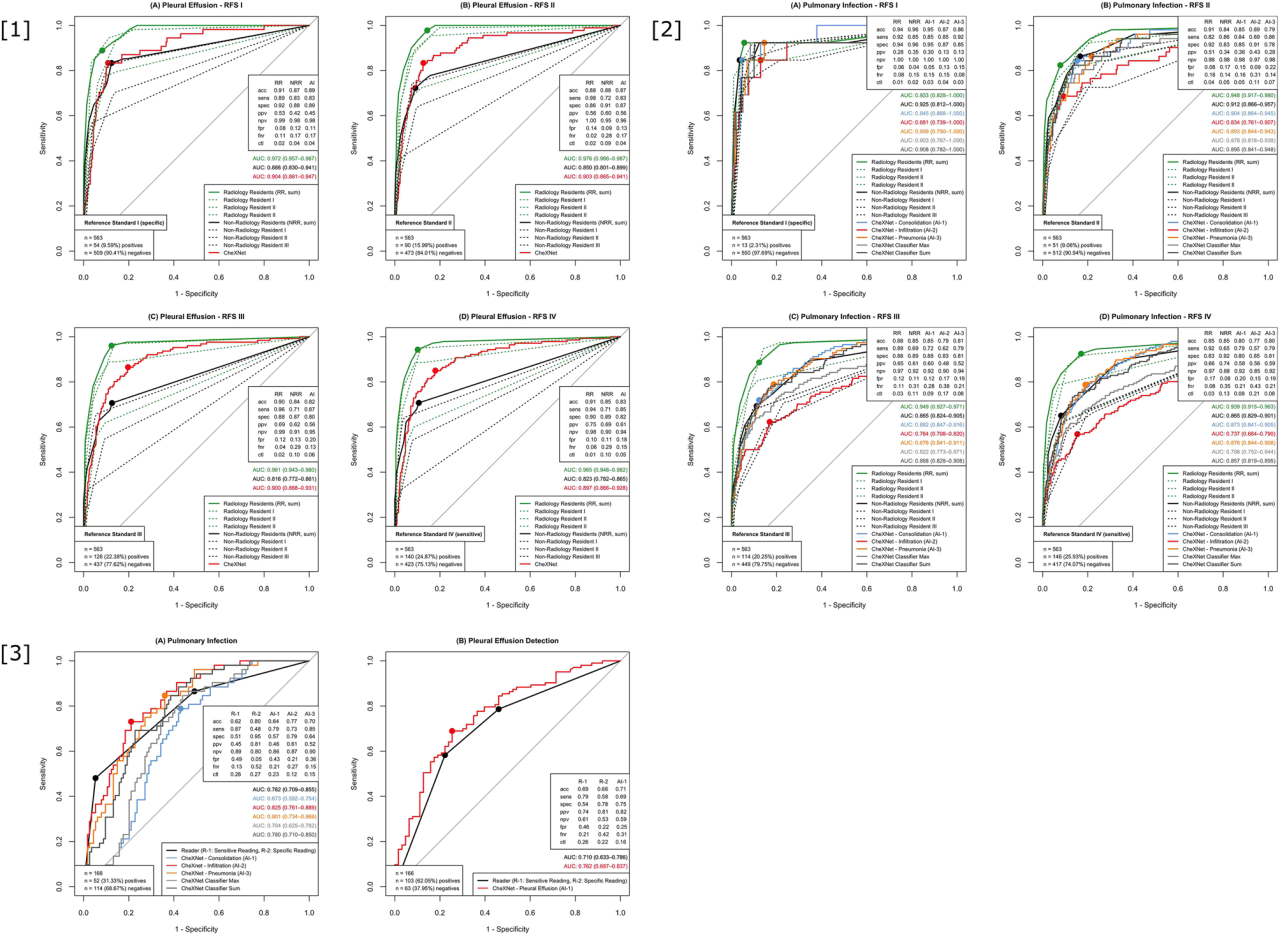
Benchmarking of pleural effusion and pulmonary infection detection [**1**]; CheXNet’s performance in pleural effusion detection (classifier “Effusion”) in the CXR EU cohort is displayed for all four RFS (RFS I-IV, (A)–(D)). CheXNet tended to perform better than NRR consensus but worse than RR consensus; [**2**] Pulmonary infection detection rate in the CXR EU cohort is displayed for all four RFS (**A**–**D**). Besides RR and NRR performance, CheXNet classifiers “Consolidation”, “Infiltration” and “Pneumonia” are pooled and displayed. Classifiers “Max” and “Sum” represent the combination of the three individual CheXNet classifiers (maximum output and sum of the outputs). In the most clinically relevant RFS IV classifiers “Consolidation” and “Pneumonia” performed on the level of NRR consensus. Classifier “Infiltration” was statistically significant outperformed by NRR and RR consensus. The combined classifiers did not outperform the individual ones; [**3**] Performance in the SCXR BLO cohort—(**A**) In pulmonary infection detection classifiers “Infiltration” and “Pneumonia” tended to exceed reader consensus’ performance (black line). Classifier “Consolidation” performed (not statistically significant) worse than the reader consensus. The combination of the three classifiers did not outperform the reader consensus—(**B**) Classifier “Effusion” performed slightly better than the reader in pleural effusion detection (not statistically significant).

Pleural effusion and consolidation benchmarking is realized with our CXR EU cohort (Fig. 3—[1] and [2]) and our SCXR BLO cohort (Fig. 3—[3]) allowing for an evaluation on both CXRs and SCXRs with BCRs’ CXR assessment (CXR EU cohort) and CT (SCXR BLO cohort) as reference standards.

Performance presentation in the CXR EU cohort is analog to PTX above. In case of pleural effusion and the clinically relevant, most sensitive RFS IV (Fig. 3—[1] (D)) CheXNet classifier “Effusion” (AUC: 0.897) performed better than the NRR consensus (AUC: 0.823, *p* < 0.01) but performed under the level of RR consensus (AUC: 0.965, *p* < 0.001). Considering the consolidations suspicious for pneumonia CheXNet (classifiers “Consolidation” and “Pneumonia”) mimicked NRR consensus in RFS IV (Fig. 3—[2](D); CheXNet classifier “Consolidation” AUC: 0.873 (*p* = 0.756), CheXNet classifier “Pneumonia” AUC: 0.876 (*p* = 0.663), NRR consensus AUC: 0.865) but was exceeded by RR consensus performance (AUC: 0.939 – comparison to “best” classifier “Pneumonia”: *p* < 0.01). CheXNet classifier “Infiltration” underperformed with an AUC of 0.737 (comparison to NRR consensus: *p* < 0.001). The combination of the three CheXNet classifiers did not outperform the individual classifiers (“Maximum” AUC: 0.798, “Sum” AUC: 0.857).

In the SCXR BLO cohort CheXNet classifiers “Infiltration” (*p* = 0.390) and “Pneumonia” (*p* = 0.710) non-significantly exceeded the radiology reader consensus for pneumonia suspicious consolidations (Fig. 3—[3] (A); CheXNet classifier “Infiltration” AUC: 0.825; classifier “Pneumonia” AUC: 0.801; reader consensus AUC: 0.782). CheXNet classifier “Consolidation” underperformed with an AUC of 0.673 and was outperformed by the classifier “Infiltration” (*p* < 0.01). Combining the three classifiers led to performances in between the individual CheXNet classifiers (“Maximum” AUC: 0.704, “Sum” AUC: 0.780). In pleural effusion detection CheXNet classifier “Effusion” non-significantly exceeded the reader’s (BCR) performance (Fig. 3—[3] (B); CheXNet classifier “Effusion” AUC: 0.762; Reader AUC: 0.710, *p* = 0.336). For comparison with originally published performance results see Table 3.

### Pulmonary lesions

**Figure 4:**
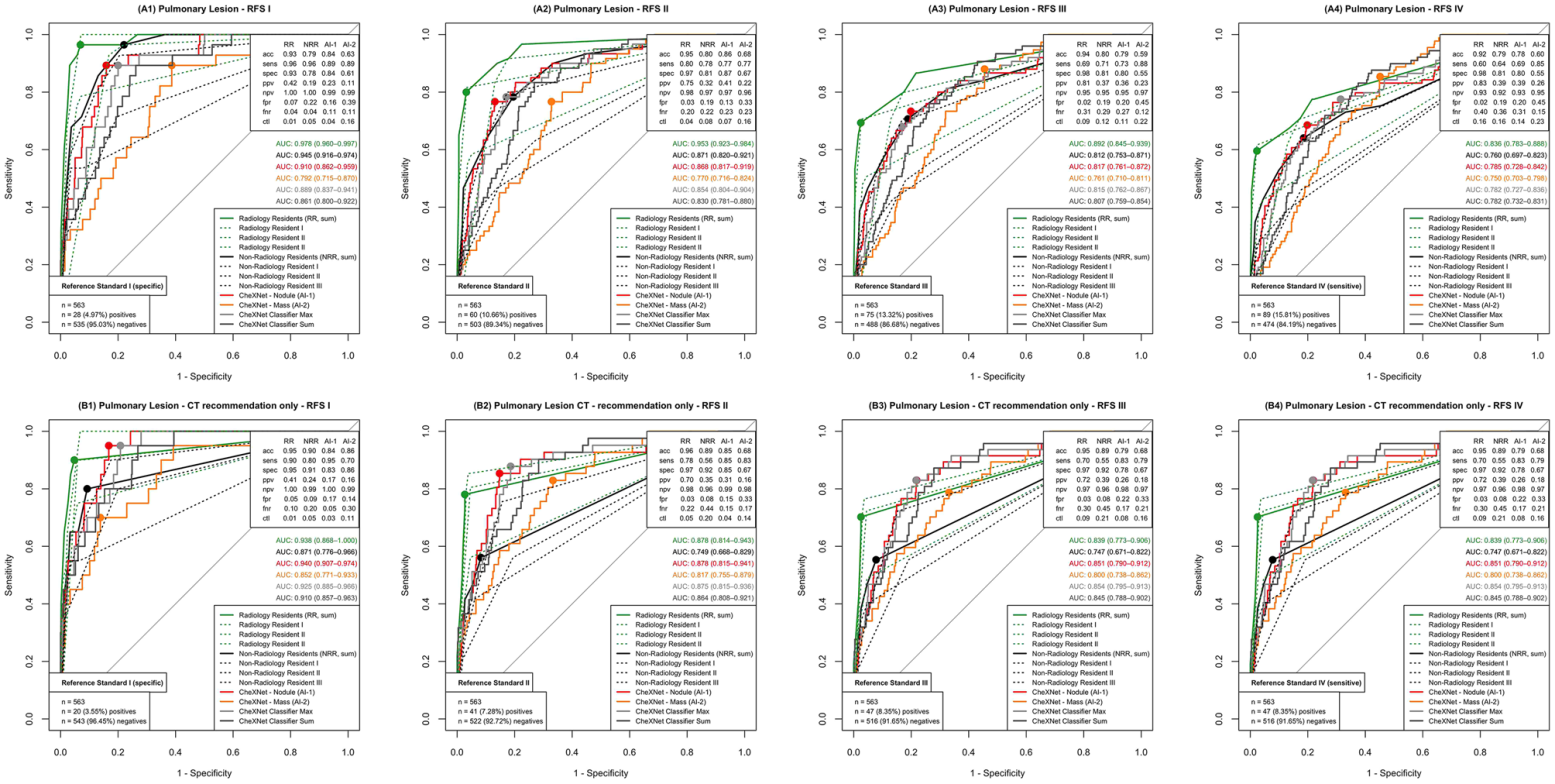
Benchmarking of (suspicious) pulmonary lesion detection; Performance results in CXR EU cohort for all four RFS (RFS I–IV) are displayed for pulmonary lesion detection in general (**A1**–**A4**) and for suspicious pulmonary lesions when CT was recommended by CXR readers (**B1**–**B4**). RR, NRR and classifiers “Nodule” and “Mass” performance is displayed as ROC curves. Classifiers “Max” and “Sum” represent the combination of two CheXNet classifiers (maximum output and sum of the outputs). In the clinically most relevant RFS IV classifier “Nodule” performed slightly better than NRR consensus (**A4**) and could even beat (not statistically significant) RR consensus AUC in the detection of the potentially suspicious pulmonary lesions (**B4**). Classifier “Mass” performed slightly better than the NRR consensus detecting potentially suspicious lesions (**B4**) but slightly underperformed NRR consensus in general lesion detection (**A4**). The combination of the two classifiers did not outperform the better performing classifier “Nodule” in general lesion detection (**A4**). Classifier “Maximum” performed slightly better than classifier “Nodule” in the potentially suspicious lesions (**B4**).

Our CXR EU cohort enables benchmarking of pulmonary nodule detection and further grading with respect to potential malignancy (Fig. 4).

Regarding pulmonary lesions in general, CheXNet classifier “Nodule” mimicked NRR consensus in the most sensitive RFS IV (Fig. 4 [A4]; CheXNet classifier “Nodule” AUC: 0.785; NRR consensus AUC: 0.760, *p* = 0.562) but was non-significantly exceeded by RR reader consensus performance (AUC: 0.836, *p* = 0.201). CheXNet classifier “Mass” performed under the level of NRR (*p* = 0.808) /RR (*p* < 0.05) consensus and classifier “Nodule” (*p* = 0.356) with an AUC of 0.750. Combining the two classifiers by considering the maximum values (“Maximum”) and the sum (“Sum”), in both cases led to an AUC of 0.782 which was lower than the AUC of the better performing CheXNet classifier “Nodule”.

Concerning the clinically most relevant potentially malignant nodules (BCR recommended a follow-up computed tomography), CheXNet classifier “Nodule” tended to perform better than the RR (*p* = 0.793) and NRR (*p* < 0.05) consensus in RFS IV (Fig. 4 [B4]; CheXNet classifier “Nodule” AUC: 0.851; RR consensus AUC: 0.839; NRR consensus AUC: 0.747). Classifier “Mass” (AUC: 0.800) showed a tendency towards better performance than the NRR consensus (*p* = 0.285), but (non-significantly) performed under the level of the RR consensus (*p* = 0.399). The combination of both classifiers resulted in AUCs of 0.854 (“Maximum”) and 0.845 (“Sum”). The “Maximum” therefore performed slightly better (not significantly significant) than individual classifier “Nodule”. For comparison with the results of the original publication see Table 3.

## Discussion

CheXNet demonstrated good performance results in the detection of suspicious pulmonary nodules with a tendency to exceed RR and NRR consensus (sum) performance (which might have a relevant clinical impact in early diagnostics), whilst exceeding the AUC of the original publication ^21^. Solid performance could be shown in the detection of pleural effusions and consolidations suspicious of pneumonia: The algorithm (non-significantly) outperformed the readers in both pathologies in the SCXR BLO cohort, showed a tendency to exceed NRR consensus for pleural effusions in the CXR EU cohort and mimicked NRR consensus in the detection of consolidations suspicious of pneumonia in the CXR EU cohort. Interestingly and potentially of a beneficial clinical impact is the good performance of the algorithm in the detection of basal pneumonia and pleural effusions in the SCXR BLO cohort which is known to be very challenging for human readers. Here, the CheXNet classifier “Infiltration” showed the most promising results. Notably, the same classifier underperformed in the CXR EU cohort in which only CXR in upright positioning were considered. This phenomenon might be explained by the annotation in the training dataset, where it was found to be often associated with atelectasis and effusions^20^. At this point, the training dataset might have used an unfavorable terminology, which has been controversially discussed^34,35^. Solid performance results throughout both cohorts were reached by the CheXNet classifier “Pneumonia” which showed better AUCs than in the original publication^21^. In pneumothorax detection, CheXNet performance showed insufficient performance results in both tested cohorts (CXR EU and SCXR PTX cohort) with smaller calculated AUCs for classifier “Pneumothorax” than originally published^21^. In the subgroup analysis of our SCXR PTX cohort, we could furthermore observe that the performance correlates positively with the proportion of inserted thoracic tubes in PTX positive images and negatively with the proportion of thoracic tubes inserted in PTX negative control images. We can therefore infer that the underlying publicly available training data for pneumothoraces was insufficient and could partially lead to a misdirected algorithm training for thoracic tubes whilst further annotations are missing. These effects have been previously presented and discussed by Rueckel et al.^31,36^.

The main strength of our study design with different benchmarking cohorts is the variability of testing different clinically relevant scenarios. The tested algorithm can run several benchmarks one after the other in a sort of benchmarking pipeline. Thus, detection rates of the different pathologies tested are not simply reported as AUC values but can be further differentiated with respect to different subgroups: Depending on patient positioning, applied reference standards, the expression of the pathology and in comparison to differently qualified radiological readers. In the following, we will highlight the advantages and disadvantages of each cohort:

The Emergency Unit Chest Radiograph Cohort (CXR EU) is a powerful cohort that compares AI performance for all the four investigated pathologies with RR and NRR reading performance using the BCR consensus as the reference standard. It is particularly distinguished by its selection exclusively of images from the emergency department, which gives it a very clinically relevant character, as these patients are usually seen for the first time. Since non-radiologists (NRR) are also involved in primary diagnostics, their performance is given special importance as a benchmarking level. Further strengths of the cohort include the high number of cases (563 images) and readers (9 readers), the strong reference standard (BCR readers experienced with up to of 17 years in thoracic imaging) and a statistical workup with different reference standards which also takes general uncertainty and different confidence levels supposedly depending on pathology extent into account. Limitations include: a single-centered reading design with RRs being trained by BCRs, preselection of the cases by an RR (potential small selection bias—clear findings might be overrepresented), case number too low to quantify possible effects of pathology co-occurrences, reader AUC can be influenced by interpolated ROC-parts (result of the rough-staged suspicion scores) and the limitation to the mentioned four pathologies.

The Supine Chest Radiograph Unilateral Pneumothorax Cohort (SCXR PTX) allows testing for weaknesses in algorithm training concerning pneumothorax detection. Its key strength is the subgroup analysis with consideration of the presence of thoracic tube and the size extent of the pneumothorax. If an algorithm was trained solely based on NLP-extracted pathology related image labels (without catheter-/tube-based image labels or in-image annotations), there is a risk that the tube (which is obviously much more prevalent in PTX positive images) is detected rather than the pleural dehiscence line itself ^31^. In a recent study, Rueckel et al.^36^ could show that these systematic errors can be partially suppressed and overall performance significantly improved if the AI system was trained with in-image annotations related to the PTX shape. Another noteworthy strength is the cohort size with a total of 6258 cases and numerous cases in every subgroup (see Table 1). Limitations of the cohort include: the single-center study design (only locally used thoracic tubes), other potential imaging confounders are not considered (e. g. other types of catheters such as central venous lines, electrocardiogram-electrodes or other nonannotated or noncontrolled image features), only supine CXR have been included (detection rates might differ in upright PA CXRs).

The Supine Chest Radiograph Basal Lung Opacities (SCXR BLO) cohort is a benchmarking cohort that addresses differentiation of basal consolidations on SCXR images, which is considered very difficult by radiologists with detection accuracies of pneumonia on SCXR being usually lower than in autopsy, bronchoalveolar lavage or CT scans^37–41^. The main strength of the cohort is that all CXR images were correlated with very timely computed tomography scans (within 90 min) which results in a high-quality reference standard. The cohort consists of a clinically very important group of mainly critically ill patients that are under continuous surveillance. Since morbidity and mortality of hospital-acquired pneumonia is very high^42–44^, early detection of consolidations suspicious for pneumonia can be of extraordinary importance. Limitations of the cohort include the small number of readers (small consensus, no detailed interrater reliability calculation possible), the small number of suspicion scores (AUC calculation of readers is influenced by the interpolation of ROC curves) and the limitation to the findings of pulmonary infection and pleural effusion.

The three cohorts have so far been limited to the detection of four relevant pathologies. Future studies need to broaden the spectrum to also evaluate the accuracy of other parameters of (S)CXR interpretation algorithms. As in this study, the focus should reflect clinical reality (e. g. different projections, different reading settings/reference standards and comparison to different reader groups).

## Conclusion

As an example of CXR interpreting AI algorithms, CheXNet shows that the primary published performance results may well differ from the results of an external validation. With our versatile multi-cohort benchmarking, we investigated multiple clinically relevant aspects that might influence algorithm performance, considering different patient positioning, different reference standards and comparison to different medical experts’ performances.

## Abbreviations

(S)CXR: (Supine) chest radiography
(S)CXRs: (Supine) chest radiographs
AI: Artificial intelligence
NLP: Natural language processing
PA: Posterior-anterior projection
EU: Emergency unit
BCR: Board-certified radiologist
RR: Radiology resident
NRR: Non-radiology resident
RFS: Reference standard
AP: Anterior–posterior projection
PTX: Pneumothorax
ROC: Receiver-operating characteristics
AUC: Area under ROC curve
ICU: Intensive care unit
Max: Maximum
acc: Accuracy
sens: Sensitivity
spec: Specificity
ppv: Positive predictive value
npv: Negative predictive value
fpr: False positive rate
fnr: False negative rate
ctl: Closest top left
TT: Thoracic tube
CI: 95% Confidence interval
